# Optimising student-led interprofessional learning across eleven health disciplines

**DOI:** 10.1186/s12909-021-02527-9

**Published:** 2021-03-15

**Authors:** Christie van Diggele, Chris Roberts, Inam Haq

**Affiliations:** 1 Faculty of Medicine and Health, The University of Sydney, Sydney, NSW 2006 Australia; 2 Faculty of Medicine and Health, Sydney Health Professional Research Education Network, The University of Sydney, Sydney, Australia

**Keywords:** Interprofessional learning, Interdisciplinary, Health, Large-scale, Assessment, Education

## Abstract

**Background:**

Provision of effective Interprofessional learning (IPL) opportunities plays a vital role in preparing healthcare students for future collaborative practice. There is an identified need for universities to better prepare students for interprofessional teamwork, however, few large-scale IPL activities have been reported. Additionally, little has been reported on disciplinary differences in student learning experience. The Health Collaboration Challenge (HCC) is a large-scale IPL activity held annually at the University of Sydney. This study sought to explore students’ experience of early participation in an interprofessional case-based learning activity, and the similarities and differences in the perceived value of interprofessional (social) learning for each discipline.

**Methods:**

In 2018, 1674 students from 11 disciplines (dentistry, oral health, nursing, pharmacy, medicine, occupational therapy, speech pathology, physiotherapy, dietetics, diagnostic radiography, exercise physiology) participated in the HCC. Students worked in teams to produce a video and patient management plan based on a patient case. Participants completed a questionnaire, including closed and open-ended items. Quantitative data were analysed using descriptive statistics. Thematic analysis was used to code and categorise qualitative data into themes. These themes were then applied and quantified at a disciplinary level to measure prevalence.

**Results:**

In total, 584/1674 (35%) of participants responded to the questionnaire. Overall, students perceived their experience to be largely beneficial to their learning and interprofessional skill development. Positive aspects included opportunities for peer learning, collaboration, networking, and understanding the different roles and responsibilities of other health professions. Negative aspects included the video form of assessment, inequity in assessment weighting across disciplines, the discipline mix within teams and lack of case relevance.

**Conclusion:**

The learning activity provided a framework for students to practice and develop their skills in interprofessional teamwork, as they prepare for increased clinical placements. Overall, students perceived their experience as beneficial to their learning and professional development early in their degree. However, they expressed dissatisfaction with the inequity of assessment weighting across the disciplines; lack of relevance of the case across disciplines; and the activity of producing a video. Further research is needed regarding the ideal number of disciplines to include in teamwork specific to a patient case.

## Background

Provision of effective Interprofessional learning (IPL) opportunities plays a vital role in preparing health professional students for future collaborative healthcare practice. Described as occurring “*when two or more professionals learn about, from and with each other to enable effective collaboration and improve health outcomes”* [1], IPL is widely acknowledged as essential to improving the quality of patient care, patient safety and service delivery [2]. Internationally, growing evidence reinforces the positive outcomes associated with IPL, with policy makers supporting the call to better prepare health professional students for collaborative future practice [3–5]. There is broad agreement that universities need to better prepare healthcare students for further professional practice, which will involve teamwork between disciplines. Compared to uni-disciplinary healthcare teams, multi-disciplinary healthcare teams are better equipped to improve patient outcomes, with input from each profession contributing to a better quality of life and improved patient safety [6]. With the aim of enhancing the knowledge, attitudes, values, skills and behaviours of collaborative health teams, universities worldwide seek to provide students with interprofessional learning (IPL) opportunities [3]. The Health Professions Accreditors Collaborative (HPAC) endorses the deliberate design of IPL activities supporting the mastery of IPL competencies through activities that are integrated into existing curriculum and are longitudinal in nature [7].

Limited research has been conducted on large-scale interprofessional learning and its impact on foundational learner outcomes [8]. Chan and colleagues (2017) reported logistical difficulties implementing a large-scale interprofessional activity involving 801 healthcare students from seven undergraduate-entry healthcare programs, and six disciplines across two Hong Kong universities [9]. A similar study by Black et al. (2016), reported on approximately 600 students from 10 disciplines involved in IPL activities based on patient safety [10]. Although evidence suggests overall, an improvement in students’ readiness to engage in interprofessional learning, it is unclear whether differences existed between disciplines in large-scale settings [9]. In addition, debate is ongoing as to the best time to facilitate interprofessional activities during a student’s degree, with research not yet convincingly displaying a negative or positive impact on the timing of introduction of interprofessional learning exposure [11].

One of the problems in design and implementation of large-scale IPL activities, is that few institutions have been able to embed a longitudinal approach that is standardised across student cohorts [12]. However, the University of Toronto has been successful in implementing a standardised approach to IPL by ensuring their IPL policy is aligned and supported by government policy. The provision of a core competency framework and necessary university, research and hospital structures have also contributed to their success [13]. However, IPL activities are often small-scale, extra-curricula activities, facilitated by individual champions with little co-ordinated, institutional support. In preparing students for the workforce, universities have struggled to create and maintain authentic IPL activities that are inclusive of entire health professional student groups. The structure of separate faculties and schools often makes shared work difficult, with IPL activities commonly presented as extra-curricular activities involving a small portion of student cohorts, that are resource intensive to implement [12]. Reeves et al. (2016) noted that a ‘bottom up’ approach fuelled purely by educators is not likely to succeed, but with the commitment from the leadership team and at a faculty level there is a much greater chance of success and sustainability of IPL initiatives [3]. It has been suggested that the responsibility of the creation of sustainable IPL initiatives lies with institutional leaders that are able to support, endorse and negotiate such activities both within and outside of the individual department [7]. However, there is a significant gap in research reporting empirical outcomes for large-scale IPL activities as part of institutional change that can inform the debate about when and where in the curriculum IPL activities are best embedded. Additionally, there is a gap in literature reporting on the differences where multiple disciplines are involved.

An opportunity to investigate these gaps in research arose within a higher education context, where a large, research intensive university in Australia has been committed to a strategy aimed at ensuring all graduating healthcare students have exposure to assessable IPL activities. The Health Collaboration Challenge (HCC) was first implemented in 2015 and included 1220 students from eight health disciplines, steadily growing to encompass larger numbers of students, and further disciplines. The HCC has been previously described [12]. The HCC is a mandatory component of the curricula for all students. In 2018, 11 healthcare disciplines participated in the HCC, developed as part of a University wide approach to deliver IPL to all health professional students. The principal features of the HCC are small IPL student teams working on a complex patient case scenario. Students collaborate to provide a patient management plan and create a short video to communicate some of their teamwork strategies. Using the conceptual framework of social constructivist theory, we sought to explore specific students’ experience of participating in the 2018 large-scale interprofessional activity.

### Theoretical framework

Theories supporting learning and teaching practices offer lenses that are helpful when considering educational methods [14]. Social constructivist theory is based on the work of Vygotsky (1978), and is centred on the understanding that learning is not individually constructed, instead mediated by the environment through joint social construction [15, 16]. The key features include:

1. Students’ knowledge transfer is facilitated by authentic tasks
2. Knowledge is continuously constructed and specific to context
3. Personal meaning is created
4. Social interaction occurs amongst learners, teachers and the environment

This theory applies to the three learning domains of IPL as outlined by Burr (2003) as the acquisition of knowledge, skills and behaviours [17]. It accentuates that when students are learning in ‘interprofessional’ teams, they are learning actively through their interactions to achieve a shared understanding of clinical practice and common goals [5]. We applied social constructivist theory to assist in interpreting the learning experiences for student interprofessional groups participating in large-scale HCC interprofessional activities, with a focus on the ways in which their professional discipline impacted their learning.

Our specific research questions were:

- How does early participation in an interprofessional case-based learning activity assist students in the development of interprofessional knowledge, skills, attitudes and behaviours?
- What are the similarities and differences in the perceived value of interprofessional (social) learning for each discipline?

## Methods

### Research context and participants

In 2018, 1674 students from 11 health disciplines [Dentistry (year 3 postgraduate), Diagnostic radiography (year 2 undergraduate, year 2 postgraduate), Dietetics (year 2 postgraduate), Exercise physiology (year 4 undergraduate, year 1 postgraduate), Medicine (year 2 postgraduate), Nursing (year 2 postgraduate), Occupational therapy (year 4 undergraduate, year 2 postgraduate), Oral health (year 3 undergraduate), Pharmacy (year 4 undergraduate, year 2 postgraduate), Physiotherapy (year 3 undergraduate, year 2 postgraduate), and Speech pathology (year 3 undergraduate, year 2 postgraduate)] took part in the HCC. Students had varying degrees of experience in interprofessional learning prior to participation.

#### Learning outcomes of the HCC activity

The student learning outcomes were to:

- Understand the contribution of a range of different health professions to meet complex patient care needs
- Integrate and prioritise key contributions from different health professions into a patient management plan
- Apply a collaborative approach to problem solving with different health professions for a challenging creative task

##### Team formation

The HCC activities required students to work in teams of five or six students, with a minimum of four different disciplines per team. Teams were allocated by faculty to ensure a mix of five to six disciplines per team. In total, 288 teams of five or six health professional students were formed.

##### HCC activity design

**Fig. 1:**
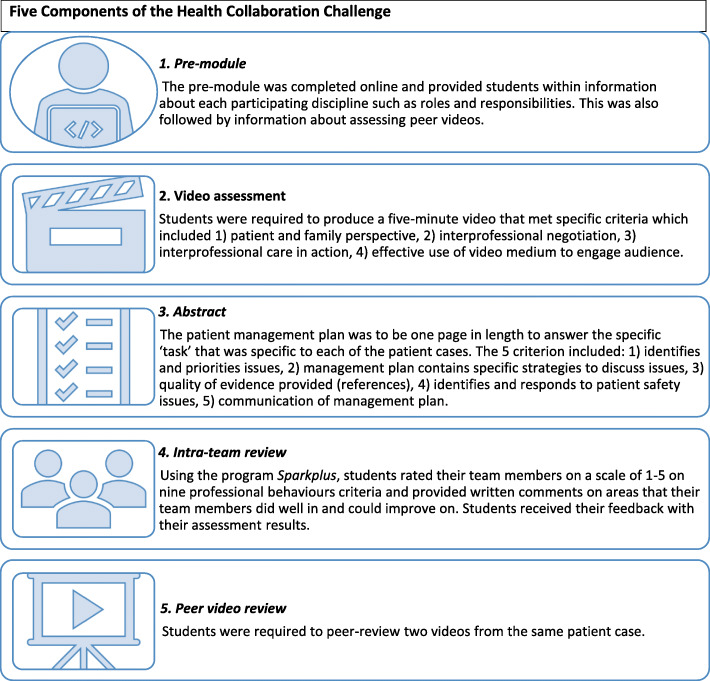
Five components of the HCC

The HCC activity required students to work in their small interprofessional teams. Student teams received one of fifteen patient cases that were refined by an interprofessional academic team to ensure their suitability for the disciplines involved. There were five components as outlined in Fig. 1. Students were required to: 1) review a complex patient case, 2) produce a 5-min video on their case management, 3) develop a one-page management plan, 4) peer review two other team videos, and 5) peer review their team members’ contribution.

A blended approach was used to deliver the HCC, via the University’s learning management system, Canvas and one face-to-face session. As the activity was student-led, students were required to complete a pre-module online before meeting their team members in order to understand how to complete the assessment tasks of a patient management plan and team video addressing the complex patient case. Students then had five days to peer review two team videos produced by other teams, and peer review their team members’ contribution.

##### Assessment and feedback

Student teams submitted two assessable components online. This included the five-minute video worth 60% and a one-page abstract worth 40% of their overall HCC assessment grade. The video submission of each team (*n* = 288) was peer assessed and the abstracts were marked by academics (*n* = 15). Rubrics were provided for each component to support consistency in marking. Written feedback was also provided to students for both components. There were differences between each unit of study regarding how the grade from this interprofessional activity contributed to students’ summative assessment. This was set by unit of study coordinators. For example, in medicine, although the HCC was a required activity, a grade did not contribute to students’ summative assessment. However, for most other disciplines the grade contributed between 5 and 20% of students’ summative assessment.

### Data collection and analysis

#### Student questionnaire

Following completion of all HCC activities, participants were invited to complete a validated online anonymous questionnaire regarding their experiences with and outcomes of the program [12]. For closed items 1 to 9, we used a five-point Likert scale ranging from ‘strongly disagree’ (1) to ‘strongly agree’ (5). Additionally, students were asked to rate the difficulty of the patient case, using a Likert scale of 1 to 5, with (1) being ‘very easy’, (2) ‘easy’, (3) ‘neither easy nor difficult’, (4) ‘difficult’, and (5) ‘very difficult’. The questionnaire also included demographic questions. Quantitative data were analysed using descriptive statistics in SPSS (version 24) [18].

To gain a greater understanding of students’ experience, we used two open ended questions: 1) ‘What was most beneficial for your learning?’ and 2) ‘How might the HCC be improved for interprofessional learning next year?’ Each response (R) was assigned an anonymous identifier (1–381) for question 1, and (1–374) for question 2. This study used a sequential design following the methodology of Miles and Huberman [19]. A thematic analysis of a sample of the qualitative data was performed within each category by two authors (CvD and CR). The coding throughout focused on the socio-cultural influences of the student experiences, interactions, and beliefs that impacted on their interprofessional learning. Codes and categories were identified inductively and translated into a coding structure. The remaining data were coded and categorised into themes by one author (CvD) which was then quantified to measure thematic prevalence [20]. These themes were then applied and quantified at a disciplinary level to determine their prevalence within each discipline (i.e. specific to dentistry, diagnostic radiography, dietetics, exercise physiology, medicine, nursing, occupational therapy, oral health, pharmacy, physiotherapy, and speech pathology).

### Ethical considerations

The University of Sydney Human Research Ethics Committee approved the study (Project number: 2015/556).

## Results

### Questionnaire response rate

In total, 584/1674 (35%) of HCC participants responded to the questionnaire. Of these respondents 405/584 (69%) were female, 149/584 (26%) were male, and 30/584 (5%) did not state their gender.

### Responses to closed items

A total of 519/584 (89%) students responded to all closed items 1–9. Of these respondents, 71/519 (14%) were nursing students, 45/519 (9%) speech pathology, 66/519 (13%) occupational therapy, 52/519 (10%) physiotherapy, 18/519 (3%) exercise physiology, 62/519 (12%) pharmacy, 71/519 (14%) medicine, 60/519 (12%) diagnostic radiography, 30/519 (6%) dentistry, 12/519 (2%) oral health, and 32/519 (6%) dietetics.

#### Student median responses to closed items

Students’ median responses are shown in Table 1. Median outcomes ≤3 (Neither Agree nor Disagree) to show where there are clear differences in responses across disciplines. Overall, students’ perception of their experience was positive with a median response of 4 for most items. Notably, nursing, speech pathology, exercise physiology and pharmacy rated the HCC activities the most highly (median score of 4 or higher for each item 1–9); while dentistry placed least value on the activities (median score 3 or less for each item 1–9). Diagnostic radiography, dentistry and oral health poorly rated the relevance of the patient case to their profession (median score 3 or less for item 1). Medicine, dentistry and oral health placed least value on the video collaboration task in comparison with all other disciplines (median score 3 or less for item 5).

**Table 1:** Participants’ (*N* = 519) median responses to their perception of the HCC activity by discipline

Discipline	N	Item 1 The patient case study was relevant to my profession	Item 2 The HCC Developed my problem-solving skills	Item 3 The HCC sharpened my analytic skills	Item 4 The HCC helped me develop my ability to work as a team member	Item 5 Collaborating on the production of a video develops teamwork skills	Item 6 Assessing the videos produced by other teams helped me gain a different perspective on case management for my scenario	Item 7 I feel confident about working with other health professions	Item 8 I will be able to use the skills and knowledge gained in my future placements	Item 9 Overall, the HCC was a worthwhile learning activity for me
Nursing	71	4	4	4	4	4	4	4	4	4
Speech Pathology	45	4	4	4	4	4	4	4	4	4
Occupational Therapy	66	4	4	3	4	4	4	3	3	4
Physiotherapy	52	4	4	3.5	4	4	4	4	4	4
Exercise Physiology	18	4	4	4	4	4	4	4	4.5	4
Pharmacy	62	4	4	4	4	4	4	4	4	4
Medicine	71	4	4	3	4	3	3	3	3	3
Diagnostic Radiography	60	2	4	4	4	4	4	4	4	3
Dentistry	30	2	3	2.5	3	2.5	2	3	2	2
Oral Health	12	3	4	3.5	4	2	4	3.5	3.5	3
Dietetics	32	4	4	4	4	3.5	3	4	4	4

(1 = Strongly Disagree, 2 = Disagree, 3 = Neither agree nor disagree, 4 = Agree, 5 = Strongly Agree)

#### Students’ perception of the difficulty of the cases based on discipline

**Fig. 2:**
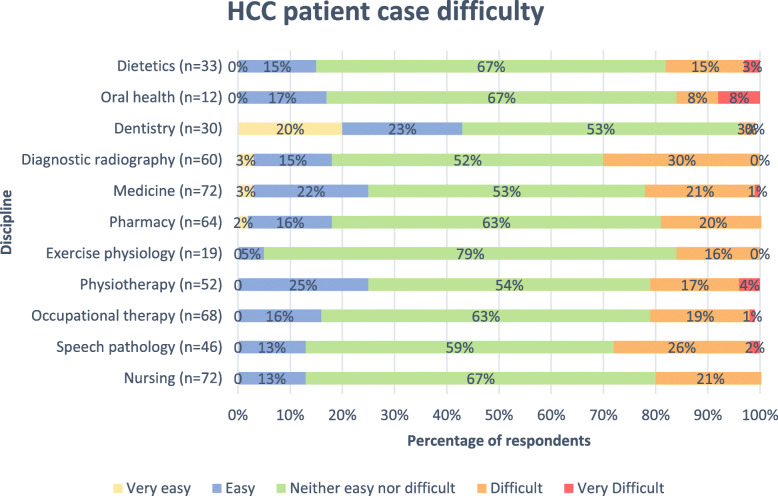
HCC 2018 patient case difficulty by discipline (*N* = 528)

Figure 2 presents students’ perception of the difficulty of the cases based on discipline. A total of 528/584 (90%) students responded to this question. Of these respondents, 72/528 (14%) were nursing students, 46/528 (9%) speech pathology, 68/528 (13%) occupational therapy, 52/528 (10%) physiotherapy, 19/528 (4%) exercise physiology, 64/528 (12%) pharmacy, 72/528 (14%) medicine, 60/528 (11%) diagnostic radiography, 30/528 (6%) dentistry, 12/528 (2%) oral health, and 33/528 (6%) dietetics. The majority of students from each discipline reported the cases as being ‘neither easy nor difficult’. However, 43% of dentistry students reported finding the case to be ‘very easy’ or ‘easy’. Notably, 28% of speech pathology students, and 30% of diagnostic radiography students found the patient case to be ‘difficult’ or ‘very difficult’.

### Responses to open-ended questions

#### Student responses: perceived benefits to learning

In total, 381/584 (66%) of respondents commented on *‘What was most beneficial to your learning?’* Of these respondents, 58/381 (15%) were nursing students, 56/381 (15%) were medical students, 40/381 (10%) were pharmacy students, 20/381 (5%) were dentistry students, 8/381 (2%) were oral health students, 32/381 (8%) were physiotherapy students, 28/381 (7%) were speech pathology students, 49/381 (13%) were occupational therapy students, 41/381 (11%) were diagnostic radiography students, 14/381 (4%) were exercise physiology students, and 25/381 (7%) were dietetic students.

Responses were categorised into six themes (Themes 1 to 6) as presented and explained in Table 2. The themes identified were:

1. Opportunity to practice working in an interprofessional team

**Table 2:** Most prevalent themes in response to the question “What was most beneficial to your learning?” (*N* = 381)

Most prevalent themes across all disciplines	Explanation of each theme	Overall theme occurrence in all disciplines (N=281)
*Theme 1:* Opportunity to practice working in an interprofessional team	Students appreciated the opportunity to practice working in an interprofessional team with students of other disciplines, with limited opportunity otherwise in the curriculum.	103 (27%)
*Theme 2:* Peer Learning and collaboration	Students valued the experience of learning from peers and collaborating with each other in their small teams.	93 (24%)
*Theme 3:* Role clarification	Students gained a better understanding of the roles and responsibilities of other health professions by taking part in the activity.	71 (19%)
*Theme 4:* Networking and socialising with other health professional students	Socialising and networking with students from other health professions was beneficial to student learning.	66 (17%)
*Theme 5:* The task itself was beneficial to student learning and provided opportunity for communication and interaction	The activity was a valuable learning experience, including comments related to the video assessment task. Students found the opportunity to further develop their communication skills and interact with others as beneficial to their learning.	63 (17%)
*Theme 6:* Perspectives of other disciplines in patient management	Students were able to see the perspectives of other health professions in the overall care of their patient	45 (12%)

For example, ‘*The teamwork component was a good opportunity to find out the scope of practice of other disciplines and to learn to interact and collaborate on a case for people who do not have previous interdisciplinary experience this would be a good learning opportunity..*’ (Nursing student, R18)

2) Peer learning and collaboration

For example, ‘*The best aspect was meeting people from other professions and talking to them about their point of view about things. Really good to collaborate.’* (Dentistry student, R3)

3) Role clarification

For example, ‘*It allowed me to learn from other disciplines and the roles of other health professionals.’* (Oral health student, R3)

4) Networking and socialising with other health professional students

For example, ‘*Getting to meet other students from various disciplines.’* (Occupational therapy student, R46)

5) The task itself was beneficial to student learning and provided opportunity for communication and interaction

For example, ‘*It is a very engaging and interactive activity. HCC was a great opportunity to have students to work together, understand and bond with other disciplines.’* (Nursing student, R9)

6) Perspectives of other disciplines in patient management

For example, ‘*Learning about the priorities of other health professionals when assessing a case.’* (Dietetics student, R19).

#### Student responses: suggested improvements for interprofessional learning

In total, 374/584 (64%) of respondents commented on ‘*How might the HCC be improved for interprofessional learning next year?*’ Of these respondents, 56/374 (15%) were nursing students, 57/374 (15%) were medical students, 36/374 (10%) were pharmacy students, 22/374 (6%) were dentistry students, 9/374 (2%) were oral health students, 29/374 (8%) were physiotherapy students, 31/374 (8%) were speech pathology students, 52/374 (14%) were occupational therapy students, 46/374 (12%) were diagnostic radiography students, 9/374 (2%) were exercise physiology students, and 27/374 (7%) were dietetic students.

These comments were characterised into six common themes (Themes 7 to 12) as presented and explained in Table 3. These additional themes identified were:

7) Equality in assessment weighting

**Table 3:** Most prevalent themes in response to the question “How might the HCC be improved for interprofessional learning next year?” (*N* = 374)

Most prevalent themes across all disciplines	Explanation of each theme	Overall theme occurrence in all disciplines (N=374)
*Theme 7* Equality in assessment weighting	Students were aware of the inequality of assessment weightings between disciplines	77 (21%)
*Theme 8* Improve the team mix and case relevance	An improvement in the discipline mix of teams and case relevance would be beneficial in managing the patient case	70 (19%)
*Theme 9* Modify the form of assessment	Students described a dislike for video assessment and suggested other forms of assessment	68 (18%)
*Theme 10* Equal contribution and time commitment	Students disliked medical students leaving early and feel that all students should contribute equally to the assessment	32 (9%)
*Theme 11* Logistical improvements and place in each curriculum	Room availability and registration for the activity could be streamlined and improved further. Select a different time within the student’s degree to have the assessment when the experience would be more relevant	32 (9%)
*Theme 12* Task guidance and assessment instructions	Students found the assessment instructions or site navigation difficult. Students would appreciate more guidance on the assessment task from facilitators on the day	16 (4%)

For example, *‘Only some team members had weighted marking for the assessment. Others did not so that affected amount of effort put towards the project’.* (Physiotherapy student, R5)

8) Improve the team mix and case relevance

For example, ‘*It would have been helpful to have the necessary personnel based on the case. For example, my group would have benefited from a dental student and occupational therapist’ (Medical student,* R14)

9) Modify the form of assessment

For example, ‘*I don’t think that creating a video that focuses on creative ways to demonstrate inter-professional learning was really the most constructive way of facilitating learning.’* (Occupational therapy student, R2)

10) Equal contribution and time commitment

For example, ‘*It was a shame that the medicine student had to leave at midday for classes as I felt he had a lot to contribute and was a great leader.’* (Diagnostic radiography student, R39)

11) Logistical improvements and place in each curriculum

For example, ‘*Do the HCC in 1st year of the masters of nutrition and dietetics instead of the 2nd. I had already done a placement that involved working in a multi-disciplinary team, so I did not gain anything from the HCC. The HCC would have been useful for the placement had it been done the other way around.’* (Dietetics student, R23)

12) Task guidance and assessment instructions

For example, ‘*More clear and concise instructions given for students, earlier in advance if possible.’ (Exercise physiology student, R8).*

### Disciplinary specific findings

#### Disciplinary specific findings: perceived benefits to learning

Disciplinary specific student responses to ‘*What was most beneficial to you learning?’* are presented in Table 4, with illustrative quotes to support our findings. The most common theme, mentioned by all disciplines, with the exception of oral health was Theme 1 ‘Opportunity to practice working in an interprofessional team’, with many students reporting this was their first time participating in an interprofessional team setting. The second most prevalent theme was Theme 2 ‘Peer learning and collaboration’, with 7/11 disciplines highlighting the value of general team collaboration and the opportunity to work in a team setting. These comments did not make reference to the interprofessional aspect of the activity. Similarly, Theme 4, ‘Networking and socialising with other health professional students’, was mentioned by 7/11 disciplines, including, medicine, dentistry, oral health, physiotherapy, occupational therapy, diagnostic radiography, and exercise physiology. Theme 3, ‘Role clarification’, mentioned by 5/11 disciplines, was common amongst pharmacy, oral health, speech pathology, occupational therapy and diagnostic radiography. Students highlighted their improved ability to better understand their own roles and responsibilities within a team, as well as the roles of others. Theme 5, ‘The task itself was beneficial to student learning and provided opportunity for communication and interaction’ was mentioned by only 2/11 disciplines, medicine and nursing. Theme 6, ‘Perspectives of other disciplines in patient management’ was mentioned only by dietetics.

**Table 4:** Theme occurrence with disciplinary quotes in response to “What was most beneficial to your learning?” (*N* = 381)

Most prevalent themes	Number and percentage of respondents	Examples of quotes from respondents
**Nursing (*N*** **= 58)**		
Theme 2: Peer learning and collaboration	(*n* = 23/58) 40%	*The teamwork component was a good opportunity to find out the scope of practice of other disciplines and to learn to interact and collaborate on a case for people who do not have previous interdisciplinary experience this would be a good learning opportunity …* (R18)
		*Collaboration and making a plan for the patient that I think was coherent, comprehensive and integrative.* (R28)
Theme 1: Opportunity to practice working in an interprofessional team	(*n* = 20/58) 34%	*The teamwork component was a good opportunity to find out the scope of practice of other disciplines and to learn to interact and collaborate on a case for people who do not have previous interdisciplinary experience this would be a good learning opportunity...* (R18)
		*Being able to work in a multidisciplinary team. It was wonderful to get to know other students from different faculties*. (R31)
Theme 5: The task itself was beneficial to student learning and provided opportunity for communication and interaction	(*n* = 10/58) 17%	*It is a very engaging and interactive activity. HCC was a great opportunity to have students to work together, understand and bond with other disciplines.* (R9)
		*I loved collaborating with the other disciplines and the use of a creative medium made it fun!* (R23)
**Medicine (*N*** **= 56)**		
Theme 5: The task itself was beneficial to student learning and provided opportunity for communication and interaction	(n = 28/56) 50%	*The best part was discussing the case with the other health professionals.* (R26)
		*It had a lot of creative freedom and discussion* (R2)
Theme 1: Opportunity to practice working in an interprofessional team	(*n* = 19/56) 34%	*Working with other healthcare students! Working through a case together was largely beneficial. Seeing what other people’s priorities were was interesting.* (R5)
		*… The importance of MDT collaboration in case management.* (R12)
Theme 4: Networking and socialising with other health professional students	(n = 19/56) 11%	*Working with other healthcare students! Working through a case together was largely beneficial. Seeing what other peoples priorities were was interesting*. (R5)
		*Interacting with other health disciplines.* (R50)
**Pharmacy (*N*** **= 40)**		
Theme 1: Opportunity to practice working in an interprofessional team	(*n* = 12/40) 30%	*I liked that we got to work with other disciplines and get a gauge of what knowledge other disciplines have/don’t have.* (R12)
		*Having the opportunity to work with other health care disciplines.* (R15)
Theme 2: Peer learning and collaboration	(*n* = 11/40) 28%	*Collaborating with other health care professionals and seeing their techniques in approaching a case scenario.* (R3)
		*Collaborating on a case.* (R34)
Theme 3: Role clarification	(*n* = 7/40) 18%	*Learning about other professions other than my own.* (R10)
		*Learn what other disciplines actually do.* (R28)
**Dentistry (N = 20)**		
Theme 2: Peer learning and collaboration	(n = 7/20) 35%	*The best aspect was meeting people from other professions and talking to them about their point of view about things. Really good to collaborate.* (R3)
		*Collaborating with colleagues from other health disciplines.* (R19)
Theme 4: Networking and socialising with other health professional students	(*n* = 6/20) 30%	*Met fellow healthcare students.* (R10)
		*Social aspect.* (R13)
Theme 1: Opportunity to practice working in an interprofessional team	(n = 3/20) 15%	*Working on a case study together with students of different disciplines was interesting and worthwhile. I can understand that the HCC is designed to allow collaboration of different professions to manage a case.* (R17)
		*Meeting and working with other disciplines.* (R20)
**Oral Health (*N*** **= 8)**		
Theme 4: Networking and socialising with other health professional students	(n = 5/8) 63%	*Meeting different professions.* (R2)
		*Meeting other students and work with them.* (R6)
Theme 3: Role clarification	(n = 3/8) 38%	*It allowed me to learn from other disciplines and the roles of other health professionals.* (R3)
		*Learning about other health professionals and what they do.* (R7)
**Physiotherapy (*N*** **= 32)**		
Theme 1: Opportunity to practice working in an interprofessional team	(n = 10/32) 31%	*Working as a MDT and learning from one another*. (R8)
		*Meeting people from different professions and analysing the case from a variety of perspectives … (*R27)
Theme 2: Peer learning and collaboration	(n = 7/32) 22%	*Collaboration with other health students* (R16)
		*Being placed in a team of other disciplines, helped me to learn the role of other disciplines in the holistic care of a patient* (R18)
Theme 4: Networking and socialising with other health professional students	(n = 3/32) 9%	*Consulting with other students on their degree and how they analyse the patient.* (R22)
		*Talking between different disciplines about how everyone would contribute to the case study.* (R24)
**Speech Pathology (N = 28)**		
Theme 3: Role clarification	(n = 10/28) 36%	*Completing the HCC allowed me to have a glimpse into how other health professionals approach a client’s health and condition. It was particularly interesting see our Physiotherapy student take over the necessary OT role and activities, as we didn’t have an OT in our group.* (R10)
		*Working with other professions as SP (speech pathologist) can be a quite isolated profession especially in community setting. Also good to know more about the professions that I might refer to or get referral from.* (R25)
Theme 1: Opportunity to practice working in an interprofessional team	(n = 7/28) 25%	*Interacting with other professionals and seeing how you all can contribute to the betterment of a patient.* (R12)
		*Working in an interprofessional team and learning about each member’s scope of practice.* (R18)
Theme 2: Peer learning and collaboration	(n = 7/28) 25%	*Collaborating with students from other disciplines in a multidisciplinary team. I was lucky to have a team of motivated and diligent students for HCC.* (R20)
		*Working collaboratively, not in just a professional manner but also through a creative manner with the video making.* (R27)
**Occupational Therapy (*N*** **= 49)**		
Theme 3: Role clarification	(*n* = 18/49) 37%	*Better clarity around roles of specific clinicians, particularly pharmacology and other non-allied health because I have had no contact with these disciplines on placements.* (R2)
		*Meeting other professions and finding out more about what they do in the field, and being able to represent my own discipline and share knowledge about our scope of practice.* (R33)
Theme 1: Opportunity to practice working in an interprofessional team	(n = 8/49) 16%	*Practice working in multi-disciplinary teams* (R7)
		*The opportunity to work with medical and dental students was extremely valuable.* (R19)
Theme 4: Networking and socialising with other health professional students	(n = 8/49) 16%	*Getting to meet other students from various disciplines* (R46)
		*Meeting new people* (R49)
**Diagnostic Radiography (*N*** **= 41)**		
Theme 1: Opportunity to practice working in an interprofessional team	(*n* = 9/41) 22%	*Working with other disciplines* (R4)
		*Working with other health professions seems to be a interesting idea.* (R31)
Theme 3: Role clarification	(n = 9/41) 22%	*Meeting students from other disciplines and learning first-hand about their roles in a patient’s care. Great perspective to see them analyse a patient’s case and discuss what they would do in their profession*. (R25)
		*It was interesting to develop more of an understanding of other fields* (R35)
Theme 4: Networking and socialising with other health professional students	(n = 8/41) 20%	*Meeting other students from other professions* (R1)
		*Meeting people from other professions* (R10)
**Exercise Physiology (*N*** **= 14)**		
Theme 2: Peer learning and collaboration	(n = 6/14) 43%	*Collaborating with others* (R3)
		*Teamwork and collaboration* (R14)
Theme 1: Opportunity to practice working in an interprofessional team	(n = 4/14) 29%	*Working with other disciplines* (R4)
		*Getting to work with different disciplines and learning about each other’s roles as an allied health professional* (R8)
Theme 4: Networking and socialising with other health professional students	(n = 4/14) 29%	*Meeting new people and understanding that priorities differ depending on your profession* (R2)
		*Meeting with other undergrad (uate) students from different disciplines.* (R12)
**Dietetics (*N*** **= 25)**		
Theme 1: Opportunity to practice working in an interprofessional team	(n = 9/25) 36%	*Working with other health professionals and discussing different elements of patient care.* (R9)
		*Experience of working with other disciplines* (R17)
Theme 2: Peer learning and collaboration	(n = 5/25) 20%	*Collaborating with other professionals and making the video.* (R6)
		*The team environment* (R21)
Theme 6: Perspectives of other disciplines in patient management	(n = 5/25) 20%	*I enjoyed seeing how other health professionals play a role in the patients care and their thought process behind their actions.* (R4)
		*Learning about the priorities of other health professionals when assessing a case.* (R19)

#### Disciplinary specific findings: suggested improvements for interprofessional learning

Disciplinary specific student responses to ‘*How might the HCC be improved for interprofessional learning next year?’* are presented in Table 5, with additional illustrative quotes to further support our findings. Theme 9, ‘Modify the form of assessment’, was the most common theme of the disciplines (9/11), including nursing, medicine, pharmacy, dentistry, oral health, physiotherapy, speech pathology, occupational therapy and dietetics. Students identified the video form of assessment as being difficult and time consuming. Theme 10, ‘Equal contribution and time commitment’, was common across six disciplines, including medicine, pharmacy, dentistry, oral health, occupational therapy, and diagnostic radiography. Students felt there was an uneven distribution of work within their teams and a lack of time commitment from some disciplines.

**Table 5:** Theme occurrence with disciplinary quotes in response to the question “How might the HCC be improved for interprofessional learning next year?” (N = 374)

Most prevalent themes	Number and percentage of respondents	Quotes from respondents
**Nursing (N = 56)**		
Theme 7: Equality in assessment weighting	(n = 27/56) 48%	*I found it was unfair that each degree had a different percentage of their grade attributed to the task. I found the students who had the HCC worth 10–15% put in far more effort than those who only required a pass or fail.* (R23)
		*It is really unfair that the HCC is worth 10% for some disciplines like nursing but carries no weightage at all for other disciplines like medicine. This causes some students to not put in any effort into doing the work. Either make it worth 10% across ALL disciplines or no weightage at all.* (R33)
Theme 9: Modify form of assessment	(n = 13/56) 23%	*The video was a bit too much to work on and we couldn’t have done it if all of us were inexperienced in making a video* (R1)
		*I don’t feel the video was beneficial to our collaboration in a health care context. I feel discussing and writing a care plan is more beneficial.* (R3)
Theme 12: Task guidance and assessment instructions	(n = 6/56) 11%	*Improve Canvas so it’s easier to find and gain access to the group page* (R14)
		*Canvas interface and instructions for accessing modules, groups, upload areas …*. (R18)
**Medicine (*N*** **= 57)**		
Theme 9: Modify form of assessment	(n = 23/57) 40%	*Have alternative options for the video.* (R6)
		*The time spent filming and editing the video, for instance, could have been used to consider a second contrasting case.* (R30)
Theme 10: Equal contribution and time commitment	(n = 10/57) 18%	*Schedule more time for medical students to be able to do it* (R29)
		*Making sure all students were at the HCC for the same amount of time. Medical students had to leave halfway, it would have been better to just allocate that amount of time for all students rather than others having to stay back.* (R52)
Theme 8: Improve the team mix and case relevance	(n = 7/57) 12%	*It would have been helpful to have the necessary personnel based on the case. For example, my group would have benefited from a dental student and occupational therapist (*R14)
		*Making sure that the case is relevant to all parties involved - assign disciplines based on case* (R2)
**Pharmacy (*N*** **= 36)**		
Theme 9: Modify form of assessment	(n = 9/36) 25%	*Not having to make a video. It was pointless and did not help us learn.* (R30)
		*The complexity of the case with no additional information was not aligned with criteria of the video assessment. I did not feel like I answered the case problems within the framework of the video.* (R8)
Theme 8: Improve the team mix and case relevance	(n = 5/36) 14%	*I feel like pharmacist is not needed in my case, Dao Chan.* (R2)
		*Better allocation of health care professionals to cases* e.g. *would’ve been helpful to have a dentist in my case. In doing this, people won’t feel redundant in their case and ensures that everyone has an important role* (R22)
Theme 10: Equal contribution and time commitment	(n = 5/36) 14%	*One student in my group can only attend 4 h (9 am - 1 pm) because he has lab on that day. It would be good if everyone in the team were free on that day.* (R17)
		*Some team members had class in the middle of the day that were compulsory thus not all team members could attend for the whole day - addressing timetable issues might mean everyone is working together not just half the team* (R24)
**Dentistry (*N*** **= 22)**		
Theme 9: Modify form of assessment	(n = 8/22) 36%	*No video. Just have the different faculties collaborate on a joint case. The video is just awkward and doesn’t help the collaborative effort* (R2)
		*Take out the 30 min video. The abstract is more than enough to assess inter-professional work. Getting graded on how creative/how well you can video edit is not very fair.* (R20)
Theme 10: Equal contribution and time commitment	(n = 4/22) 18%	*Make sure med students are available for the entire day. Having to leave at 12 made it difficult particularly for groups who registered later* (R1)
		*Ensuring med students have their schedules cleared. Only available until 12 was not appropriate particularly for those groups that registered later.* (R22)
Theme 11: Logistical improvements and place in each curriculum	(n = 3/22) 14%	*Could be given to younger year (DMD2) as I feel like they would have learnt more and gotten more out of it*. (R5)
		*Have degrees not already working in hospitals with teams participate. Those whom already work in a hospital setting contacting different clinicians should be exempt as this provides no gain from what we learn in the first few weeks in a hospital setting*. (R14)
**Oral Health (N = 9)**		
Theme 10: Equal contribution and time commitment	(n = 5/9) 56%	*Medical student participation for the entire day* (R7)
		*Not everyone has this assignment graded (medicine) and they said they (medicine) had something to do and leave early but you see them eating next door relaxing and not working with us.* (R8)
Theme 9: Modify form of assessment	(n = 2/9) 22%	*Instead of a video, we should make something else as it was hard to make a video as a group.* (R3)
		*Not a video - time restraints and too hard to organize a time outside of course work.* (R9)
Theme 7: Equality in assessment weighting	(n = 2/9) 22%	*Everyone getting marked equally - some students only had to participate to pass, and therefore did not participate in all areas of the HCC assignment.* (R5)
		*I do not understand how is it possible that some students are marked and some are not. - the mark from this challenge is going towards my final mark within the unit. This is unfair for obvious reasons, just to mention two: motivation, distribution of the workload.* (R6)
**Physiotherapy (*N*** **= 29)**		
Theme 11: Logistical improvements and place in each curriculum	(n = 6/29) 21%	*Honestly, taking part on the HCC when I had completed almost 4 clinical placements was not a helpful experience. I had treated cases similar to the one presented at the HCC during placements, and done that within a MDT.* (R10)
		*I think the HCC was not relevant to those in their final years of their degree.* (R18)
Theme 9: Modify form of assessment	(n = 6/29) 21%	*The video was a waste of time. The value of HCC is from talking to other professionals about the case, not making a short clip* (R19)
		*The video aspect was very time consuming and in my opinion unnecessary. I would have preferred that we create a longer abstract and peer review each others?* (R20)
Theme 7: Equality in assessment weighting	(n = 3/29) 10%	*Only some team members had weighted marking for the assessment. Others did not so that affected amount of effort put towards the project.* (R5)
		*Don’t make the mark count for some degrees but not others. It should be unweighted for everyone.* (R6)
**Speech Pathology (*N*** **= 31)**		
Theme 9: Modify form of assessment	(n = 7/31) 23%	*The video making - it really disadvantages those groups where everyone has no experience in this.* (R26)
		*Get rid of the video aspect, a powerpoint or abstract is enough.* (R30)
Theme 12: Task guidance and assessment instructions	(n = 6/31) 19%	*Easier video submission process.* (R5)
		*Make the modules more accessible and less complex - it was extremely hard to navigate everything.* (R6)
Theme 8: Improve the team mix and case relevance	(n = 5/31) 16%	*Make sure that the case study contains relevant details for each profession.* (R15)
		*There were some team members that couldn’t really be involved as their disciplines weren’t relevant to the case,* e.g. *radiographer for me case. It would be good to only assign people to cases that they can take part in using their disciplinary knowledge.* (R7)
**Occupational Therapy (*N*** **= 52)**		
Theme 7: Equality in assessment weighting	(n = 23/52) 44%	*Equal or no weighting for all disciplines.* (R6)
		*Ensure that the HCC is a weighted % for each team member - as individuals who only need to attend and ‘pass’ may not be willing to put in as much effort as individuals who have HCC as a 20% weight in one of their subjects.* (R8)
Theme 10: Equal contribution and time commitment	(n = 17/52) 33%	*Better organisation: med students had to leave for class halfway through* (R1)
		*To ensure that all students have the day off - the med students left early and the rest of us had to stay and complete the work* (R23)
Theme 9: Modify form of assessment	(n = 9/52) 17%	*I don’t think that creating a video that focuses on creative ways to demonstrate inter-professional learning was really the most constructive way of facilitating learning* (R2)
		*I don’t think making a video is necessary, I think only a written component is necessary for students to gain same benefits from HCC.* (R45)
**Diagnostic Radiography (*N*** **= 46)**		
Theme 8: Improve the team mix and case relevance	(n = 24/46) 52%	*Our group didn’t have a medical student. We were limited due to this and the case study was irrelevant for my profession. I did not play a major part in the discussion due to this.* (R8)
		*Ensuring that individuals are assigned to patient case studies that are more relevant to their profession* (R4)
Theme 10: Equal contribution and time commitment	(n = 12/46) 26%	*It was a shame that the medicine student had to leave at midday for classes as I felt he had a lot to contribute and was a great leader.* (R39)
		*Pick a date where the medical students don’t need to leave early for class if possible.* (R9)
Theme 7: Equality in assessment weighting	(n = 9/46) 20%	*Ensuring that the HCC has a balanced assessment weighting across disciplines can improve the contribution of each team member. I was fortunate to have hard working team mates, but I heard that other teams encountered difficulties because some members didn’t contribute as the assessment wasn’t marked for them, which is unfair for others*. (R43)
		*Make sure it is graded for all involved. It was graded for me, but what a pass/fail for everyone else in the group, so I feel like I had to take on more responsibility.* (R41)
**Exercise Physiology (N = 9)**		
Theme 11: Logistical improvements and place in each curriculum	(n = 5/9) 56%	*Better signage for finding learning spaces at RPA. There was a construction zone in front of the building that made it hard to find.* (R5)
		*Have the HCC at Cumberland campus, as that’s where the majority of health science degrees are located. (R4)*
Theme 12: Task guidance and assessment instructions	(n = 4/9) 44%	*Information released earlier so we have a better understanding of what is expected early on and can better prepare for it. (R1)*
		*More clear and concise instructions given for students, earlier in advance if possible (R8)*
**Dietetics (N = 27)**		
Theme 11: Logistical improvements and place in each curriculum	(n = 9/27) 33%	*Less rigidity regarding timetable* (R11)
		*Do the HCC in 1st year of the masters of nutrition and dietetics instead of the 2nd. I had already done a placement that involved working in a multi-disciplinary team, so I did not gain anything from the HCC. The HCC would have been useful for the placement had it been done the other way around.* (R23)
Theme 9: Modify form of assessment	(n = 4/27) 15%	*The video felt more like a chore than a learning activity.* (R26)
		*Group debrief (larger group) on different case perspectives eg. discussion/healthy debate.* (R10)
Theme 8: Improve the team mix and case relevance	(n = 4/27) 15%	*From speaking to other students, some were allocated a case where their profession did not have a significant role whist that role would be more beneficial in another case.* (R17)
		*Please consider incorporating a more substantial role for the radiographer.* (R22)

Theme 7, ‘Equality in assessment weighting’, was identified by 5/11 disciplines including nursing, oral health, physiotherapy, occupational therapy and diagnostic radiography. Students felt that all students should be given the same assessment weighting as they were all completing the same tasks. Theme 8, ‘Improve the team mix and case relevance’, was also identified by 5/11 disciplines, including medicine, pharmacy, speech pathology, diagnostic radiography and dietetics. It was reported that some patient cases lacked relevance to some professions. Theme 11, ‘Logistical improvements and place in each curriculum’, was prevalent in 5/11 disciplinary groups, including dentistry, physiotherapy, exercise physiology and dietetics. Theme 12, ‘Task guidance and assessment instruction’, was the least prevalent (3/11), only identified by nursing, speech pathology and exercise physiology students.

## Discussion

This study sought to explore the similarities and differences of students’ learning experience of participating in a large-scale interprofessional activity, held across 11 disciplines. Overall, participants perceived their experience to be largely beneficial to their learning and interprofessional skill development. Positive aspects included opportunities for peer learning and collaboration, the ability to learn the roles and responsibilities of different health professions, and networking and socialising with other health professions. Students were most dissatisfied with the video task as a form of assessment, the inequity in assessment weighting across disciplines, and the perceived imbalance of student contributions to teamwork. Students felt that improvements could be made to the discipline mix in teams and the relevance of case studies to the health professions of their team. Notably, dentistry was the least satisfied with the HCC activity, and reported finding the patient case less challenging. In order to consider the implications of our findings we use the categories of social constructivist theory [16], ‘student knowledge transfer’, ‘knowledge construction’, ‘personal meaning’ and ‘social interactions’ to discuss our findings, and assist our understanding of students’ interprofessional learning within the HCC activities.

### Students’ knowledge transfer is facilitated by authentic tasks

Learning transfer occurs when students apply their knowledge in a relevant context [24]. The team activities were based around the use of authentic patient cases. While our results indicate that students generally felt the patient cases had the right level of difficulty, a large proportion (43%) of dentistry students reported finding the case “very easy or easy”. Additionally, responses to open ended questions indicate that a fifth of students (19%) felt their team’s patient case lacked relevance to their own discipline. While literature highlights the importance of including the disciplinary background and knowledge of all team members when designing patient cases for small group activities [12], our results highlight the logistical difficulties of conducting interprofessional learning activities simultaneously for such a large number of disciplines [21]. Similar findings were reported by Lochner et al., 2018, who reported on an IPL activity on the common topic of ‘patient safety’, involving 39 students from five healthcare disciplines [22]. Although not a large number of students were involved, their results suggested that student learning outcomes were hindered by the excessive number of disciplines (five) working on the one patient case, with some students being afforded more valuable learning opportunities specifically relating to their disciplines [22]. Perhaps by reducing the number of disciplines included within each student team (for example, from five to three disciplines), it would be possible to increase the relevance of the case for every discipline, increasing student engagement with IPL. This would be logistically achievable by closely considering curriculum alignment for each discipline with the patient case topic, and grouping students accordingly.

### Knowledge is continuously constructed and specific to context

Learning that provides an emphasis on active learner engagement, with information created in various ways reinforces student learning [23, 24]. The HCC assessment tasks were designed to provide a shared goal and means of engagement for all team members. Literature suggests that the ‘peer pressure’ placed on individuals working in small groups encourages students to complete tasks [25]. However, 18% of students indicated a dislike of the video activity, and suggested a modified form of assessment. Additionally, 21% of students, predominantly from five disciplines (nursing, oral health, physiotherapy, occupational therapy and diagnostic radiography) indicated their dissatisfaction with the inequity across disciplines in the weighting of assessments on grades, with a nursing students stating *“This causes some students not to put in any effort into doing the work …*” . Chan et al. (2017) mentioned similar findings, where the IPL activity was considered integral to only four of their seven programs involved and was considered to impact on student motivation [9]. This highlights the need for consistency in the management and alignment of assessment tasks to ensure fairness and transparency for students. Buhse et al. 2017, reported in an IPL activity involving disciplines nursing, physician assistant and public health, reported the difficult challenge of creating student ‘buy in’ [26]. They anticipated that this would have been achieved by increasing the weight of summative assessment for all disciplines, indicating that without appropriate assessment weighting, the IPL activity was viewed as an ‘add on’, rather than integrated within the respective curriculum [26]. It is clear, the assessment grading needs to be changed to ensure equity across disciplines, and ensure student accountability and buy in.

### Personal meaning is created

Students play an active role in the joint creation of their own learning by taking part in meaningful learning experiences that are authentic and relatable to the ‘real world’ [27]. Our findings demonstrate that the HCC activities based on patient cases fostered students’ learning about each other’s roles in patient management, with 9% of students commenting that the activity helped them to gain an understanding of the roles of others and themselves, and 12% stating they gained an understanding of the perspective of disciplines in patient management. IPL activities in small groups allow students to construct their understanding of new knowledge and build on their attitudes and beliefs while working through problem-solving activities as a team [28]. Interprofessional activities allow for clarification of student’s own roles within a team setting, their purpose and how they can contribute to a patient/client’s care plan [4, 29, 30]. Notably, students from oral health (38%), occupational therapy (37%), speech pathology (36%), diagnostic radiography (22%), and pharmacy (18%) valued the opportunity to work with students of other health professions to further develop their understanding of roles and responsibilities of others.

Again, this stresses the importance of better alignment in interprofessional activities with individual discipline curricula. Students could see value in their disciplinary knowledge and the need for their active contribution in case management. Those that were not able to contribute or noticed that others could not contribute to the case due to the team mix or case relevance identified this as an area needing improvement. A large proportion of diagnostic radiography (52%), speech pathology (16%), dietetics (15%), pharmacy (14%), medicine (12%) felt that the case was not aligned with the roles and responsibilities of all disciplines in the team.

### Social interaction occurs amongst learners, teachers, and the environment

Learning occurs in the process of meaning construction, involving verbal interaction, questioning and negotiation of team members [17]. Twenty seven percent of students indicated that the HCC activities provided the opportunity to practice working in interprofessional teams. Although controversial, our findings support literature indicating that early experience of IPL within the first two years of health professional education enhances students’ preparedness for further interprofessional learning and improves their attitudes to interdisciplinary teamwork [31, 32]. Students (11%) felt that the small group activities of the HCC provided opportunities for communication and interaction to foster skills in listening and shared decision making among disciplines. Students need to practice working together in teams [5, 33], and the HCC activity, utilises small group work, helping to facilitate this learning process. Due to the number of students and disciplines, there was limited access to facilitators. Students wanted more guidance and feedback on the task from facilitators (4%) with some mentioning they would like more explicit training on communicating with other disciplines before attending the activity. In the junior years, there is limited opportunity for formal interactions with other disciplines [34]. Evidently, students appreciated the opportunity for this early teamwork and networking.

### Strengths and limitations

The strength of this study is that it demonstrates the feasibility and effectiveness of a large-scale IPL activity. The qualitative analysis gives a rich appreciation of the experiences of IPL, but may not be generalisable to other educational settings. The response rate is 35% and there is variation in disciplinary response rates. This may be due to distribution of the questionnaire being two weeks after the face-to-face component of the activity. Bias may exist in that those who chose to respond may have favoured the activity and our results may not be reflective of the wider population.

## Conclusion

We used social constructivist theory as a lens to interpret students’ perceptions of their experience in the HCC. By analysing the responses of students at a disciplinary level we were able to explore the perceived similarities and differences of learning benefits and improvements in interprofessional learning across 11 health professions. The learning activity provided a framework for healthcare students to practice and develop their skills in interprofessional teamwork, self and peer review and feedback, as they prepare for increased clinical and community placements. Overall, students perceived their experience to be beneficial to their learning and professional development early in their degree. Importantly, students perceived an increased understanding of the roles of other health professionals. However, some students expressed dissatisfaction with the inequity of assessment weighting across the disciplines; the lack of relevance of the case to some disciplines; and with the required activity itself, of producing a video. To build on the success of the large-scale HCC in the early years, multiple IPL activities should be offered in the senior years of healthcare education and contextualised to meet the needs of disciplines. Further research is needed in respect to disciplinary findings regarding the ideal number of disciplines to include in teamwork specific to a patient case.

## Data Availability

Datasets supporting the conclusions of this article are included within the article. Additional data at the level of individual students is not available as per confidentiality agreements approved by the Human Research Ethics Committee, University of Sydney.

## Abbreviations

HCC: Health Collaboration Challenge
IPL: Interprofessional Learning
